# Supplementary material for: Integrated Transcriptome Analysis Reveals the Crucial mRNAs and miRNAs Related to Fecundity in the Hypothalamus of Yunshang Black Goats during the Luteal Phase
Source: Animals (Basel). 2022 Dec 2;12(23):3397. doi: 10.3390/ani12233397 (PMC9738480; doi:10.3390/ani12233397)
Supplement: Supplementary file 1 [file animals-12-03397-s001.zip › Supplementary Table captions.pdf]

Supplementary Material

Supplementary Table S1 | The overall genes expressed and the fragments per kilobase per million mapped fragments (FPKM) values and chromosomes distribution of identified mRNAs identified in high fecundity goats in the luteal phase (LP-HF) versus low fecundity goats in the luteal phase (LP-LF).

Supplementary Table S2 | Differentially expressed genes identified in high fecundity goats in the luteal phase (LP-HF) versus low fecundity goats in the luteal phase (LP-LF).

Supplementary Table S3 | The overall miRNAs expressed identified in high fecundity goats in the luteal phase (LP-HF) versus low fecundity goats in the luteal phase (LP-LF).

Supplementary Table S4 | The identification involving diverse RNAs and length distribution of small RNA in high fecundity goats in the luteal phase (LP-HF) versus low fecundity goats in the luteal phase (LP-LF).

Supplementary Table S5 | Differentially expressed miRNAs identified in high fecundity goats in the luteal phase (LP-HF) versus low fecundity goats in the luteal phase (LP-LF).

Supplementary Table S6 | The list of potential target genes of differentially expressed miRNAs in high fecundity goats in the luteal phase (LP-HF) versus low fecundity goats in the luteal phase (LP-LF).

Supplementary Table S7 | GO enrichment annotation for mRNAs in terms of their molecular function (MF), biological process (BP), and cellular component (CC) level in high fecundity goats in the luteal phase (LP-HF) versus low fecundity goats in the luteal phase (LP-LF).

Supplementary Table S8 | KEGG enrichment annotation for mRNAs in high fecundity goats in the luteal phase (LP-HF) versus low fecundity goats in the luteal phase (LP-LF).

Supplementary Table S9 | GO enrichment annotation for potential target genes of differentially expressed miRNAs in terms of their molecular function (MF), biological process (BP), and cellular component (CC) level in high fecundity goats in the luteal phase (LP-HF) versus low fecundity goats in the luteal phase (LP-LF).

Supplementary Table S10 | KEGG enrichment annotation for potential target genes of differentially expressed miRNAs in high fecundity goats in the luteal phase (LP-HF) versus low fecundity goats in the luteal phase (LP-LF).

Supplementary Table S11 | The intersected gene list between DEGs and predicted target genes of DEMs in high fecundity goats in the luteal phase (LP-HF) versus low fecundity goats in the luteal phase (LP-LF).

Supplementary Table S12 | Construction of miRNA-mRNA Interaction Network in high fecundity goats in the luteal phase (LP-HF) versus low fecundity goats in the luteal phase (LP-LF).

Supplementary Table S13 | Real-time quantitative PCR (RT–qPCR) validation of differentially expressed mRNAs and miRNAs in high fecundity goats in the luteal phase (LP-HF) versus low fecundity goats in the luteal phase (LP-LF).
